# Twin differences in the Minnesota Trust Game relate to neural mechanisms of suspiciousness

**DOI:** 10.3758/s13415-025-01324-x

**Published:** 2025-07-10

**Authors:** Rebecca Kazinka, Anita N. D. Kwashie, Danielle N. Pratt, Iris Vilares, William G. Iacono, Sylia Wilson, Angus W. MacDonald

**Affiliations:** 1 Psychiatry Department, University of Minnesota Medical School, Minneapolis, MN USA; 2 Biomedical Engineering Department, University of Minnesota, Minneapolis, MN USA; 3 Psychology Department, University of Minnesota, Minneapolis, MN USA; 4 Psychology Department, Northwestern University, Evanston, IL USA; 5 Institute for Child Development, University of Minnesota, Minneapolis, MN USA

**Keywords:** Twin study, Computational model, Decision-making, Schizophrenia, Reward

## Abstract

Spite sensitivity, or the fear that a person is willing to intentionally take a loss to ensure that another person will as well, may be a key component in understanding persecutory ideation (the belief that others want to harm you). We implemented a co-twin control design to examine potentially causal relationships among persecutory ideation, spite sensitivity, and neural activity and connectivity. Sixty-nine participants (23 monozygotic twin pairs and an additional 23 unpaired monozygotic twins) completed the Minnesota Trust Game—a social decision-making game played asynchronously with an anonymous partner that targets spite sensitivity by varying the incentives of the partner. Participants with more self-reported persecutory ideation (relative to those with lower persecutory ideation) trusted less even when the partner was incentivized to be fair. Similarly, computational modeling showed that increased persecutory ideation was associated with greater beliefs of a partner’s spitefulness. Twins with greater beliefs of a partner’s spitefulness (relative to their co-twins) also reported higher persecution. In addition, twin differences in left lateral OFC activation during the task were associated with spite sensitivity. These results point towards a potentially causal role of the lateral OFC on spite sensitivity, and in turn effects of spite sensitivity on persecutory ideation.

**Supplementary Information:**

The online version contains supplementary material available at 10.3758/s13415-025-01324-x.

## Introduction

Although debilitating psychotic disorders affect a relatively low proportion of the population (~ 1%), attenuated psychotic-like experiences are far more common (Kendler et al., 1996; Johns & Van Os, 2001). Persecutory delusions, beliefs that others are conspiring against oneself, exist at the extreme; however, many people endorse questions suggestive of persecutory ideation, such as “I often feel that others have it in for me” (Bebbington et al., 2013; Verdoux & Van Os, 2002; van Os and Reininghaus 2016). The variability in endorsement of persecutory ideation suggests a spectrum of paranoia or excessive mistrust or suspicion of people’s willingness to hurt you, annoy you, or conspire against you (Freeman & Loe, 2023). In individuals with psychosis, persecutory ideation has been associated with theory-of-mind deficits, such that they have difficulty understanding another’s intentions, leading to conflicts in relationships (Mehl et al., 2010; Langdon et al., 2005). Even in nonclinical populations, persecutory ideation is associated with social anxiety and fear of others’ negative evaluations, which make social relationships more fraught (Martin & Penn, 2002). In the present paper, we aimed to 1) assess social decision-making and related neural functioning in individuals who report elevated levels of persecutory ideation yet are not diagnosed with a psychotic disorder, and 2) leverage differences between participants and their monozygotic co-twins to provide insight into the potentially causal impacts of persecutory ideation on interpersonal behaviors and related brain activation.

Social decision-making paradigms, in which choices affect not only the player but also others, can reveal not just an individual’s preferences about a tangible reward, such as money, but also their sense of fairness and beliefs about a partner’s intentions (Alos-Ferrer & Farolfi, 2019). Traditional trust games have sequential choices between two players, such that the first mover’s monetary offer is multiplied when given to the second mover, and the second mover decides how much money to return. Importantly, the second mover is disincentivized to return any money (i.e., earn the most winnings), but often participants pay some amount back (Berg et al., 1995). However, trust games often only assess situations when the second mover has reasons to be untrustworthy. Our study uses the Minnesota Trust Game (MTG), which expands upon traditional trust games by controlling the amount of money each player can receive to set up two different conditions based on the second mover’s incentives (Johnson et al., 2009). Like the traditional trust game, in one condition the second mover is offered two choices, and taking the higher amount leads to a loss for the first mover (Rational Mistrust); we extend the traditional game by including a condition in which the second mover receives less money if they choose to give the first mover less money as well, incentivizing the second mover to be trustworthy (Suspiciousness). This small change in the incentives in the MTG allows us to target spite sensitivity, or the fear that the second mover will purposefully choose a poor outcome for themselves to ensure that the first mover suffers as well. The incentive structures in the MTG can uniquely provide insights into concerns that another person may behave spitefully in the Suspiciousness condition; we leverage this feature to target spite sensitivity’s relationship with persecutory ideation.

The triple network model has been used to explain dysfunction of neural networks in psychopathology, suggesting that balance of the central executive network (CEN; also called the FPN) and default mode network (DMN; including the OFC, vmPFC) is modified by the salience network (including the caudate nucleus) (Botvinick et al., 2001; Menon, 2011). In psychosis, the triple network model may be impaired in terms of aberrant salience, in which the brain assigns undue attention to irrelevant internal and external stimuli (Kapur, 2003; Howes et al., 2020). Aberrant salience, particularly related to subcortical regions associated with dopaminergic function, has been implicated in delusion formation (Miyata, 2019). Furthermore, a previous study of first-episode psychosis participants demonstrated hypoconnectivity for both salience-central executive networks and salience-default mode networks, suggesting the failures of salience network extended to the CEN and DMN (Kim et al., 2022). Noise in both CEN and DMN, and particularly failure of the CEN to regulate the intrusions in the DMN, occurs in psychosis and contributes to positive symptoms (Looijestijn et al., 2015). This network model provides a useful framework to understand possible dysfunction related to persecutory ideation.

Prior research in the MTG has found that individuals higher in persecutory ideation are less trusting in the Suspiciousness condition, even though they are incentivized to trust (Johnson et al., 2009; Wisner et al., 2021). In addition, there is mixed evidence that persecutory ideation is associated with computational measures of spite sensitivity (Kazinka et al., 2022; 2024). Neuroimaging results from the first mover’s perspective while playing the MTG match similar results seen in traditional trust games related to mistrust and expand our understanding of neural correlates of spite sensitivity. Individuals with psychosis have shown reduced caudate nucleus activity during a Rational Mistrust condition, increasing the player’s perceived risk of losing money, similar to results in the trust game (Gromann et al., 2013). However, in the Suspiciousness condition, there was greater lateral orbitofrontal cortex (OFC) activity associated with increased risk of losing money for the first mover (Kazinka et al., 2024). Of note, this brain region, which falls into the salience network, is thought to play a role in context-specific coding of inferred outcomes (Qiu et al., 2024) and integrating information from multiple sources to guide behavior (Tegelbeckers et al., 2023). We hypothesize that dysfunction of this region may contribute to failures to predict context-specific outcomes, contributing to persecutory ideation. In addition, specific functional connectivity patterns correspond to suspicious beliefs in individuals with psychosis, namely decreased connectivity between frontoparietal regions (FPN; a part of the CEN) and the ventromedial prefrontal cortex (vmPFC)/OFC (Wisner et al., 2021); a part of the DMN). Kazinka et al. (2024) further found that dysconnectivity between the FPN and the OFC/salience network was associated with spite sensitivity in individuals with psychosis. These findings indicated different neural correlates when predicting rational mistrust versus suspicious mistrust, suggesting that they are separate constructs.

In this study, we had the unique opportunity to implement the co-twin control design (McGueet al., 2010), which is a common method used in twin research (Boomsma et al., 2002; Neale & Cardon, 1992). While most past research on spite sensitivity has focused on cross-sectional designs that are limited to correlational relationships, the co-twin control design can provide additional insights into potentially causal mechanisms. Monozygotic twins have shared genetics and many shared experiences; by comparing an individual against their co-twin, we can control for these factors. This approach allows us to compare differences in traits within a pair, which represent unshared experiences. When these differences also predict differences in measured outcomes, then we can more confidently assert that the differences indicate a causal relationship or indirect shared confound with the measured outcomes. While it does not necessarily include longitudinal information that would strengthen arguments for causal inference, it suggests a stronger causal relationship than simply correlations within unrelated populations. For example, the co-twin control design has suggested potential causal influences linking persecutory ideation to symptoms, such as hallucinations, cognitive disorganization (Zavos et al., 2014), and depression (Zavos et al., 2016). Of note, the MTG has previously been studied in a separate small sample of monozygotic twins without psychosis using the co-twin control design (Wisner et al., 2021). Wisner & colleagues replicated the relationship between FPN-vmPFC/OFC disconnectivity and persecutory ideation seen in individuals with psychosis and further found that co-twin differences in persecutory ideation were associated with differences in trust in the Suspiciousness condition, suggesting that persecutory ideation may be causally linked to trust in the Suspiciousness condition (i.e., spite sensitivity). The co-twin control model can provide stronger evidence for the role of spite sensitivity in persecutory ideation.

The current study examines spite sensitivity in a community sample selected to match the range of self-reported suspiciousness found in an early psychosis sample previously reported (Kazinka et al., 2024). We additionally recruited a subsample of their monozygotic co-twins to examine potentially causal associations of spite sensitivity with persecutory ideation. We aimed to replicate previous results, but we were most interested in examining twin differences to further tease apart the potentially causal role of spite sensitivity and neural activation during the MTG on persecutory ideation. The co-twin control design provides stronger evidence that a priori brain regions are associated with spite sensitivity and that spite sensitivity is predictive of persecutory ideation, providing valuable insights for future research on treating persecutory ideation.

## Methods

### Participants

Seventy-two individual monozygotic twins were recruited from the ES and MS cohorts from the Minnesota Center for Twin and Family Research (MCTFR) at the University of Minnesota (Keyes et al., 2009; Wilson et al., 2018; Iacono et al., 2006). Participants were between 18 and 45 years. In this study, we were interested in nonclinical levels of persecutory ideation. Exclusion criteria included DSM-IV diagnosis of a mental disorder with psychosis, diagnosis of a substance use disorder in the past 3 months, current intoxication, health issues contraindicating MR scanning, history of neurological or developmental disorders or traumatic brain injury, or an estimated IQ < 70 as determined by the Wechsler Test of Adult Reading (WTAR) (Wechsler, 2001). Forty-nine of these individuals (referred to as probands) were selected based on having a score on the Multidimensional Personality Questionnaire (MPQ) Alienation subscale that could be near-matched to scores for individuals with psychosis previously described by Kazinka et al. (2024), providing us with a community sample with elevated MPQ-Alienation but not diagnosed with psychosis. In total, three probands were excluded (for autism spectrum disorder, bipolar disorder, or claustrophobia), leaving 46 eligible probands (Table 1). We recruited a subsample of 23 co-twins of the probands (referred to as “co-twins”), not selected for their scores on MPQ-Alienation. Participants were not specifically discordant on psychotic symptoms to provide more variance in our analysis. Procedures for this study were approved by the University of Minnesota institutional review board.

**Table 1:** Sample demographics

Category	Participants	Relationship with MPQ- Alienation	Relationship with spite-guilt beliefs
N	69	69	69
Age	30.9 (5.3)	Estimate = −.15, SE =.197, *t* =.8, *p* =.426	Estimate = −.04, SE =.04, *t* =.948, *p* =.348
% Male	43.5%	t(44) = 0.76, *p* =.451	t(44) = 1.27, *p* =.211
Years of Education	16.5 (2.3)	**Estimate = − 1.34, SE =.402, *t* = 3.34, *p* =.002**	Estimate =.06, SE =.1, *t* =.64, *p* =.525
Parental Years of Education (average)	14.8 (3.2)	Estimate = −.495, SE =.310, *t* = 1.59, *p* =.119	Estimate =.029, SE =.073, *t* =.403, *p* =.689
Handedness Laterality	73.3 (55.8)	Estimate = −.02, SE =.017, *t* = 1.19, *p* =.24	Estimate = −.001, SE =.004, *t* =.171, *p* =.865
% Racial Minority	2.9%	W = 91, *p* =.4	W = 28, *p* =.168
% Hispanic or Latinx	1.4%	W = 19, *p* =.466	W = 49, *p* =.466
MPQ-Alienation	34.5 (8.0)	-	**Estimate = − 1.45, SE =.48,** ***t* = 3.06, *p* =.005**

### Personality and clinical measures

The Multidimensional Personality Questionnaire (MPQ) is commonly used to assess a range of personality measures and consists of 300 questions scored 1 − 4 on a Likert scale (Patrick et al., 2002; Tellegen & Waller, 2008); a subsample of scales from the MPQ were administered to all participants. Most notably, persecutory ideation was measured using the MPQ-Alienation subscale (Patrick et al., 2002; Tellegen & Waller, 2008), which assesses feelings that others will harm them, that others cannot be trusted, and of betrayal (18 questions; Cronbach’s alpha = 0.86) (Patrick et al., 2002). This scale has been used to assess paranoid beliefs previously and has been shown to be elevated in psychosis samples (Johnson et al., 2009; Wisner et al., 2021; Kazinka et al., 2022; Arseneault et al., 2000; Brennan et al., 2024). To screen for mental health symptoms on the assessment day, trained research assistants administered the Depression, Mania, Alcohol Use, Substance Use, and Psychosis sections of the Mini International Neuropsychiatric Interview 5th edition (M.I.N.I., interrater ICC-2 = 0.8) (Sheehan et al., 1998). Handedness laterality was assessed using the Edinburgh Handedness Inventory (Oldfield, 1971), in which positive values reflect right-handedness and negative values reflect left-handedness.

### Decision-making task

**Fig. 1:**
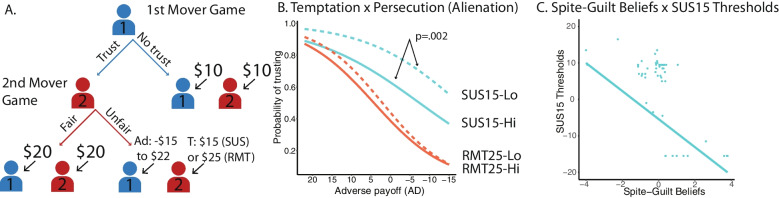
Schematic of Minnesota Trust Game. **A)** Decision tree for the two roles in the Minnesota Trust Game: First Mover and Second Mover. The first mover decided whether to take a small sum of money (S = $10) or let the second mover decide between two options: one where both partners got a mutually beneficial larger amount of money (M = $20) or an uneven choice. Temptation T was manipulated to either be greater than M (Rational Mistrust—RMT25) or less than M (Suspiciousness—SUS15). Adapted from Kazinka et al., 2024 and used with permission. **B)** Aggregate first-mover behavior across all community participants, divided by condition. Overall, trust decreases as the adverse payoff decreases, yet participants are generally more trusting in the Suspiciousness condition. Individuals who were higher on MPQ-Alienation were less likely to trust particularly during the Suspiciousness condition (shown as a median split for clarity). **C)** We replicated a significant negative relationship between spite-guilt beliefs and thresholds in the Suspiciousness condition. Line fit represents the predicted values from the regression model

The Minnesota Trust Game (MTG) is a social decision-making game that depends on the decisions of the player and an anonymous partner (Fig. 1). There are two sequential roles: the first mover decides between two choices (take a small amount of money or trust the second mover to decide); if given the opportunity, the second mover decides between two different ways of allocating money to the first mover and to themselves. Importantly, there is no immediate feedback in the game, so these roles were played asynchronously. Participants played as the second mover first and then reversed roles. Our results focused on the perspective of the first mover. This paradigm is designed to distinguish between sources of distrust, depending on the partner’s motivations (either incentivized or disincentivized to betray the first mover). First movers decide to either take a small, safe amount of money for both players ($10) or choose to let the second mover decide between two options that are known to the first mover. The second mover’s choices would then be between a mutual option with an equal, larger amount of money for each player ($20) or unequal amounts where the second mover receives some temptation (T), and the first mover receives some adverse payoff (AD). There are two conditions based on the temptation: when T = $25, the second mover is incentivized to betray the first mover to get a larger reward (because T is greater than $20), which we refer to as the Rational Mistrust condition (RMT25); when T = $15, the second mover is *disincentivized* to betray the player (receiving less than $20), which we refer to as the Suspiciousness condition (SUS15)*.* Adverse payoffs vary in each trial from losing $15 to winning $22 to parametrically identify a boundary between trusting and not trusting (referred to as thresholds). Payment incentives were determined by a random trial from each game based on the participant’s choices paired with the choices of a random previous participant.

### Data analysis

#### Behavioral analyses

Analyses were conducted in RStudio (v. 4.2.2) unless otherwise specified. Behavior on the MTG was assessed by calculating a threshold for each condition (RMT25 and SUS15) using a Heaviside function to identify where participants switch between trusting and not trusting in the MTG. Higher thresholds indicate greater mistrust. To test the hypothesis that individuals with greater persecutory ideation (compared with those with less) were less trusting in the SUS15 condition but not the RMT25 condition, we conducted a mixed-effects logistic regression. The model predicted the choice to let the second mover decide (i.e., trust the second mover) or take the safe payoff ($10), based on condition (RMT25 or SUS15), persecutory ideation (MPQ-Alienation) and adverse payoff (each AD value offered) nested within family (i.e., twin pairs). To examine spite sensitivity, we calculated the first mover’s percentage of choices to not trust the second mover during the SUS15 condition (referred to as suspicious mistrust). We conducted similar analyses from the second mover’s decision, which can be found in the supplementary materials (Fig. S4).

We additionally applied a computational model previously tested and validated on two separate samples (Kazinka et al., 2022), using principles from the Fehr-Schmidt inequity aversion model (Fehr & Schmidt, 1999) and reported on a sample of individuals with psychosis (Kazinka et al., 2024). This model calculates four parameters: 1) envy (⍺), or the dislike of a partner’s larger outcome; 2) estimated partner spite-guilt (β'; referred to as spite-guilt beliefs, a measure of spite sensitivity), which measures the estimation of the partner’s guilt about a greater outcome (for positive values) or spitefulness towards the player (for negative values); 3) risk aversion (*R*), which assesses the difference of the adverse payoff to the safe amount; and 4) lambda (λ), which calculates inverse temperature (i.e., noisiness in responses). We used the fmincon function in MATLAB (2018 edition) to calculate the best parameter values for each individual using maximum likelihood estimation. We hypothesized that spite-guilt beliefs would be more closely related to SUS15 condition thresholds, while risk aversion would be more associated with the RMT25 thresholds. Finally, we used a random effects linear regression to test the relationship of spite sensitivity (measured by suspicious mistrust and the spite-guilt beliefs parameter) and persecutory ideation, with family as a random effect. More details of the model are reported in the supplemental materials and in Kazinka et al. (2022).

#### Co-twin control analysis

To further examine the potentially causal effect of spite sensitivity on persecutory ideation, we conducted co-twin control analyses with 23 twin pairs, which accounts for shared familial confounds (Begg & Parides, 2024). In brief, we conducted linear mixed models using the twin pair average score (between-pair) and the twin pair difference score (within-pair) on spite sensitivity. The between-pair score controls what is shared by twins in a twin pair (i.e., shared genetic and environmental factors), while the within-pair score signifies what is unique to each twin (i.e., nonshared environmental factors). In this model, within-pair scores have positive values (where one twin is above the twin pair mean) and negative values (where the other twin is below the twin pair mean), such that a significant association suggests that the differences between the twins predict the variable of interest while controlling for family-level differences (between-pair score). This model does not incorporate longitudinal information, yet can still identify potentially causal inferences, in which the variable either directly causes the outcome or the two share an indirect causal factor. Our analyses focused on the within-pair results for behavioral results, such as thresholds and parameters, personality traits, such as MPQ-Alienation, and z-scored beta values from neuroimaging regions of interest (ROIs). We hypothesized that there would be a negative relationship, in which twins who believed the second mover would be more spiteful (negative spite-guilt beliefs) compared to their co-twins’ beliefs about spitefulness would be more likely to also have higher persecutory ideation. Standardized beta estimates are reported and can be interpreted as small (0.1 to 0.29), medium (0.3 to − 0.49), or large (> 0.5) effects.

#### Neuroimaging analyses

We conducted three task-fMRI scans of the first mover in the MTG. We additionally collected structural scans (T1) and used TOPUP (Anderson et al., 2003; Smith et al., 2004) to implement distortion correction. Two of the probands were excluded for poor resolution of the T1 structural scan. For another two of the probands, only two of the three runs were used for neuroimaging results due to movement > 0.5 mm for more than 20% of the run. One co-twin was excluded from the neuroimaging data collection because of pregnancy; another co-twin only completed two of the three scans.

Neuroimaging analyses were conducted using BOLD GLMs and functional connectivity using FSL version 6.0.4 (Jenkinson et al., 2012). The general linear model compared conditions (RMT25 and SUS15) and risk (no, low, and high risk) based on the adverse payoff. “No risk” was any adverse payoff above $10 (≥ *S*), “low risk” was an adverse payoff between $3 and $10, and “high risk” was an adverse payoff below $3. We examined contrasts only between no and high risk, the two conditions, and their interaction. We additionally assessed individual differences by using a priori ROIs from the interaction seen in individuals with psychosis (Kazinka et al., 2024), which included the bilateral caudate nucleus, left and right lateral OFC, dorsomedial prefrontal cortex (dmPFC), and ventromedial prefrontal cortex (vmPFC) (Fig. S5). We used these ROIs to compare previous results from individuals with psychosis to this community sample. We did not include any third-level covariates in the whole-brain analyses. GLMs additionally included explanatory variables for missed responses, standard motion parameters, and confound variables for timepoints in which motion was greater than 0.5 mm. We used FSL’s easythresh function with a threshold of Z = 3.1 and *p*_*brainwise*_ = 0.05. Group comparisons of the twins vs. co-twins are reported in the supplement (Fig. S6).

Additionally, we used the Spite Sensitivity computational model to calculate the subjective value of the two choices for the participant for each trial. From these calculations, we conducted a GLM that modeled the subjective value of the chosen outcome. For the trust choices, values were dependent on the adverse payoff, temptation value, and parameter estimations, while no trust choices were valued at $10 (as there were no differences between the two participants). We removed one participant from this analysis who chose to trust for 100% of decisions.

Additional GLMs are reported in full in the supplement: 1) Comparisons of the two conditions in which adverse payoff is used as a continuous variable instead of creating levels of risk, and 2) comparisons of the choice to trust or not trust for each condition.

To assess functional connectivity, we performed dual regression using FSL, which first regresses a spatial map of networks into a subject’s data set to acquire a time course and then regresses those time courses into a 4D dataset to get a subject-specific spatial map of the networks (Beckmann et al., 2009; Nickerson et al., 2017). Dual regression was applied to spatial maps of networks defined by independent components analysis from a sample of healthy community members (*N* = 218) previously reported (Rueter et al., 2018) (Fig. S9). Five networks of interest were selected: OFC, OFC/insula/dmPFC network, bilateral FPN, and caudate nucleus, based on previous studies (Wisner et al., 2021). Time courses during the suspiciousness condition from the intrinsic connectivity networks selected were correlated to calculate interconnectivity. We examined how interconnectivity between these regions related to individual difference measures (MPQ-Alienation, suspicious mistrust, and spite-guilt beliefs), and additionally examined potential causal relationships using the co-twin control design described above.

## Results

Sample demographics and their relationship to MPQ-Alienation and spite-guilt beliefs are reported in Table 1. We found that those with greater MPQ-Alienation also had fewer years of education. We therefore statistically controlled for this factor when conducting the analyses including MPQ-Alienation. Comparisons between probands and co-twins, as well as probands whose twins were recruited versus not, are reported in Table S1. Of note, there were more left-handed paired probands versus unpaired probands, but no other group differences.

### Behavioral performance and computational modeling

Minnesota Trust Game performance showed that participants had a similar pattern of behavior to previous MTG performance, in which they were more trusting in the SUS15 condition than the RMT25 condition. Logistic regression showed a significant interaction between condition and adverse payoff (β = 0.442, SE = 0.33, *Z* = 13.5, *p* < 0.001), in which participants trusted more in the SUS15 condition, compared to the RMT25 condition, as the adverse payoff decreased. There were also significant main effects of condition and adverse payoff (*p* < 0.001). These results did not change when controlling for education, which was also a significant, independent predictor (β = 0.64, SE = 0.26, *Z* = 2.43, *p* = 0.015).

We further tested the role of persecutory ideation on MTG performance. When adding persecutory ideation to the model, we found a significant three-way interaction (condition × adverse payoff × Alienation: β = 0.099, SE = 0.036, *Z* = 2.71, *p* = 0.007; Fig. 1B). We replicated a significant interaction of condition and Alienation, such that those with higher persecutory ideation were less trusting in the SUS15 condition (β = 0.141, SE = 0.034, *Z* = 4.20, *p* < 0.001). There was a main effect of persecutory ideation as well (β = − 0.92, SE = 0.054, *Z* = 16.8, *p* < 0.001). When controlling for education, the interaction of condition and persecutory ideation held. The main effect of education was not significant (β = 0.106, SE = 0.076, *Z* = 1.39, *p* = 0.163).

**Fig. 2:**
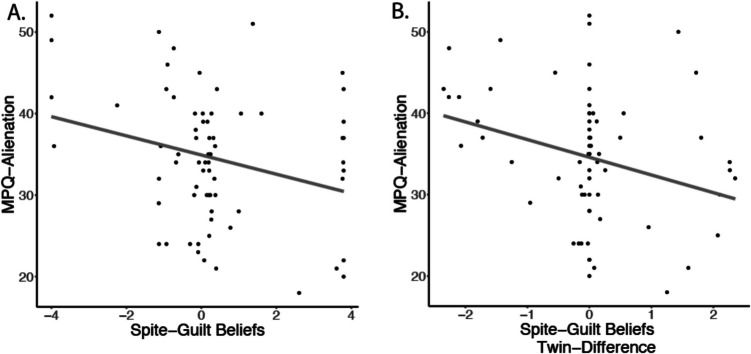
Spite-Guilt Beliefs predicts MPQ-Alienation. **A)** Spite-guilt beliefs had a strong negative relationship with MPQ-Alienation, in which more beliefs that others were spiteful was associated with reporting more alienation. **B)** The co-twin control model further supported this result by showing that twin differences in spite-guilt beliefs predicted MPQ-Alienation, suggesting a potentially causal role of spite-guilt beliefs on MPQ-Alienation. Line fit represents the predicted values from the regression model

Spite sensitivity was tested using computational modeling of the first mover. Participants showed good replication of the group data after simulating the data based on the estimated variables (Fig. S1). There was a negative relationship between spite-guilt beliefs and SUS15 condition thresholds (β = − 0.632, SE = 0.095, *t* = 6.84, *p* < 0.001; Fig. 1C), suggesting that participants trusted more when they believed the second mover would feel more guilty. We also found a strong negative correlation between spite-guilt beliefs and the RMT25 condition thresholds (β = − 0.808, SE = 0.074, *t* = 10.91, *p* < 0.001). In terms of risk aversion, we found this parameter correlated with both the RMT25 condition thresholds (β = 0.544, SE = 0.105, *t* = 5.18, *p* < 0.001) and the SUS15 condition thresholds (β = 0.377, SE = 0.113, *t* = 3.33, *p* = 0.001). Finally, we examined individual differences related to persecutory ideation, which showed a significant negative correlation with spite-guilt beliefs (β = − 0.342, SE = 0.115, *t* = 2.97, *p* = 0.005; Fig. 2A) and a positive correlation with suspicious mistrust (β = 0.33, SE = 0.116, *t* = 2.88, *p* = 0.005). These results did not differ when controlling for education. Parameter recovery was fair to excellent (*r's* > 0.56). In addition, the spite-guilt beliefs parameter was negatively correlated with risk aversion (β = − 0.340, SE = 0.142, *t* = 2.39, *p* = 0.021) (see supplement for more details; Figs. S2 and S3).

### Twin differences in behavior

To further assess the potentially causal role of spite sensitivity on persecutory ideation, we compared the selected community sample to their monozygotic co-twins in a subsample of 23 pairs. A negative correlation was found between twin differences in spite-guilt beliefs and persecutory ideation (β = − 0.29, SE = 0.09, *t* = 3.38, *p* = 0.003; Fig. 2B), so that twins with greater spite-guilt beliefs (compared with their co-twins) also had higher persecutory ideation. Results held when controlling for education. There was also a medium negative correlation between twin differences in spite-guilt beliefs and suspicious mistrust (estimate = − 0.317, SE = 0.08, *t* = 4.20, *p* < 0.001), such that twins with higher spite-guilt beliefs also had less trust during the SUS15 condition. However, we did not identify a sufficiently strong relationship between twin differences in suspicious mistrust and persecutory ideation (estimate = 0.149, SE = 0.102, *t* = 1.46, *p* = 0.158).

### Neuroimaging results

#### Main effects of risk and condition

**Fig. 3:**
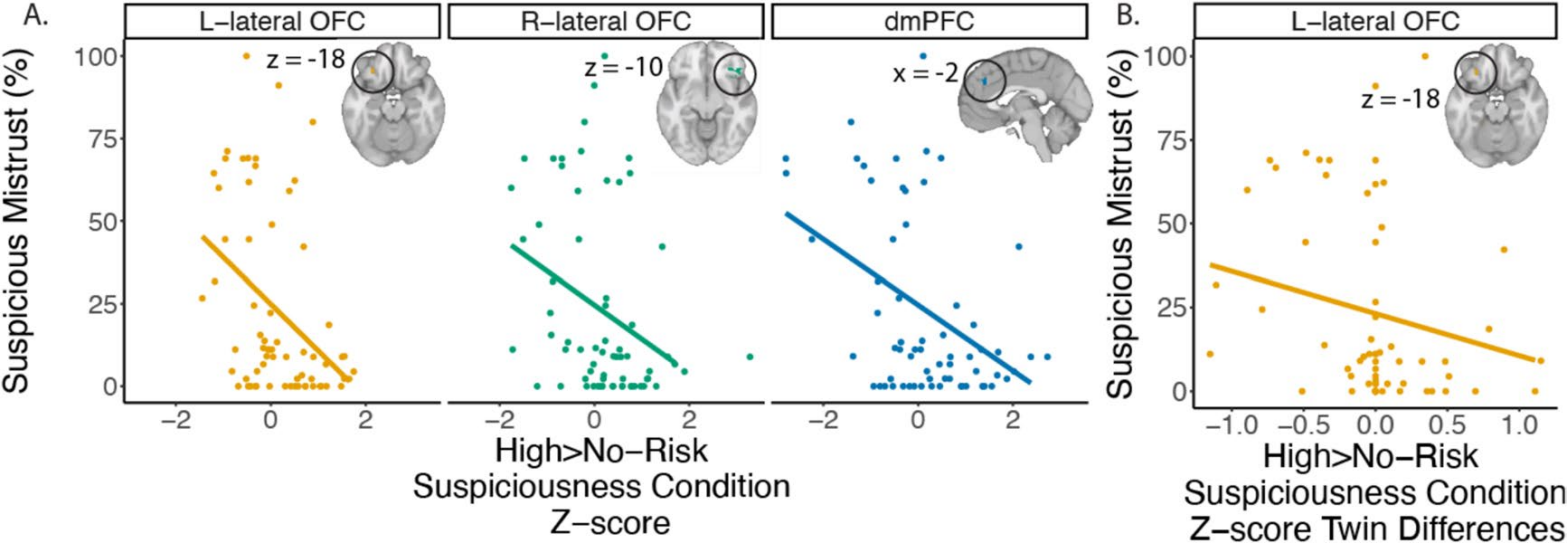
Task-based BOLD response predicts suspicious mistrust. **A)** The percentage of mistrust in the Suspiciousness condition was related to activation in the bilateral OFC and dmPFC when comparing high risk > no risk trials in the Suspiciousness condition. **B)** We further showed that twin differences in the left lateral OFC contrast of high risk > no risk trials in the Suspiciousness conditions predicted the percentage of mistrust in the Suspiciousness condition (suspicious mistrust), strengthening our understanding of this relationship. Line fit represents the predicted values from the regression model

We compared neural activation across the two conditions (RMT25 and SUS15) and two risk levels (high and no risk), based on the adverse payoff (Fig. 3). A priori ROIs included bilateral caudate nucleus, bilateral OFC, dmPFC, and vmPFC (Figs. 3A and S5). The main effect of condition (SUS15 > RMT25) showed a significant correlation between the right lateral OFC activation and suspicious mistrust (estimate = 0.278, SE = 0.127, *t* = 2.18, *p* = 0.033). When looking at high risk > no risk trials in the SUS15 condition, this contrast was negatively correlated with suspicious mistrust in the left lateral OFC (estimate = − 0.404, SE = 0.112, *t* = 3.60, *p* < 0.001), right lateral OFC (estimate = − 0.311, SE = 0.115, *t* = 2.70, *p* = 0.01), and dmPFC (estimate = − 0.368, SE = 0.114, *t* = 3.25, *p* = 0.002; Fig. 3A). This result suggests that lateral OFC played a role in choosing to trust during the SUS15 condition specifically, consistent with individuals with psychosis.

When we examined the main effect of risk, we found a significant reduction in activation in bilateral caudate during high risk trials, consistent with previous findings. In addition, there was a significantly greater activation in the no risk trials than high risk trials during RMT25 in the bilateral caudate nucleus. Activation comparing no risk > high risk during RMT25 was negatively correlated with the risk aversion parameter in the left caudate (estimate = − 0.289, SE = 0.115, *t* = 2.50, *p* = 0.015). These results are consistent with previous findings in individuals with psychosis (Kazinka et al., 2024). However, the whole brain interaction between risk and condition only showed a small region of positive activation in the caudate, showing that the caudate nucleus had a greater involvement in decision-making in the MTG in this community sample. There were minimal differences when examining twins vs. co-twins for any of the contrasts, with only small differences in the lateral occipital lobe when comparing risk in the Rational Mistrust condition. Whole brain analyses are reported in the supplemental results (Fig. S6). Whole-brain analysis of risk and condition using the adverse payoff as a continuous variable found similar results and is reported in the supplement (Fig. S7).

#### Subjective value of the chosen outcome

**Fig. 4:**
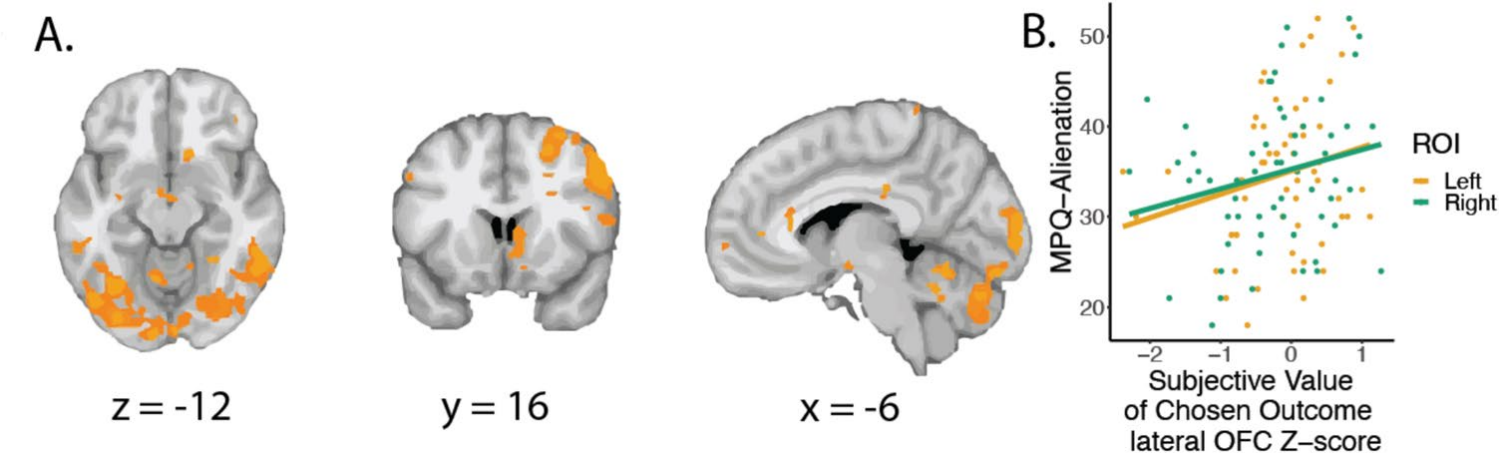
Subjective value representations are related to persecutory ideation. **A)** When we examined brain regions associated with the subjective value of the chosen outcome, we found it was associated with the right ventral striatum, right lateral OFC, right supramarginal gyrus, and the occipital lobe.** B)** Activation in the lateral OFC was correlated with persecutory ideation (MPQ-Alienation)

We examined differences in the activation of bilateral OFC and dmPFC during trust and no trust decisions and found greater activation in the bilateral OFC in trust decisions (Fig. S8). When looking across individuals, we found that activation in the left lateral OFC tracked the choice made. We ran an additional GLM examining the results of the subjective value of the chosen outcome using the Spite Sensitivity model across both conditions (Fig. 4). We found in the whole-brain analysis that the subjective value of the chosen outcome was most associated with the ventral striatum and right lateral OFC but not left lateral OFC. We examined both the left and right OFC ROIs from the main text in a linear mixed model. The lateral OFC predicted MPQ-Alienation (β = 0.197, SE = 0.094, *t* = 2.1, *p* = 0.042), but not spite-guilt beliefs, risk aversion, SUS15 thresholds, nor RMT25 thresholds (*p* > 0.062).

## Twin differences in neural activation

We additionally examined the potentially causal role of spite sensitivity on neural activations. In the SUS15 condition high risk > no risk analyses, we found that twin differences in left lateral OFC activation predicted suspicious mistrust (estimate = − 0.227, SE = 0.09, *t* = 2.53, *p* = 0.019; Fig. 3C) so that twins with more negative left lateral activation also trusted less during the SUS15 condition. These results were not seen in twin differences of the right lateral OFC or dmPFC, nor when examining relationships with spite-guilt beliefs or MPQ-Alienation. We did not see a relationship between the twin difference in the lateral OFC subjective value representation and MPQ-Alienation.

### Functional connectivity

**Fig. 5:**
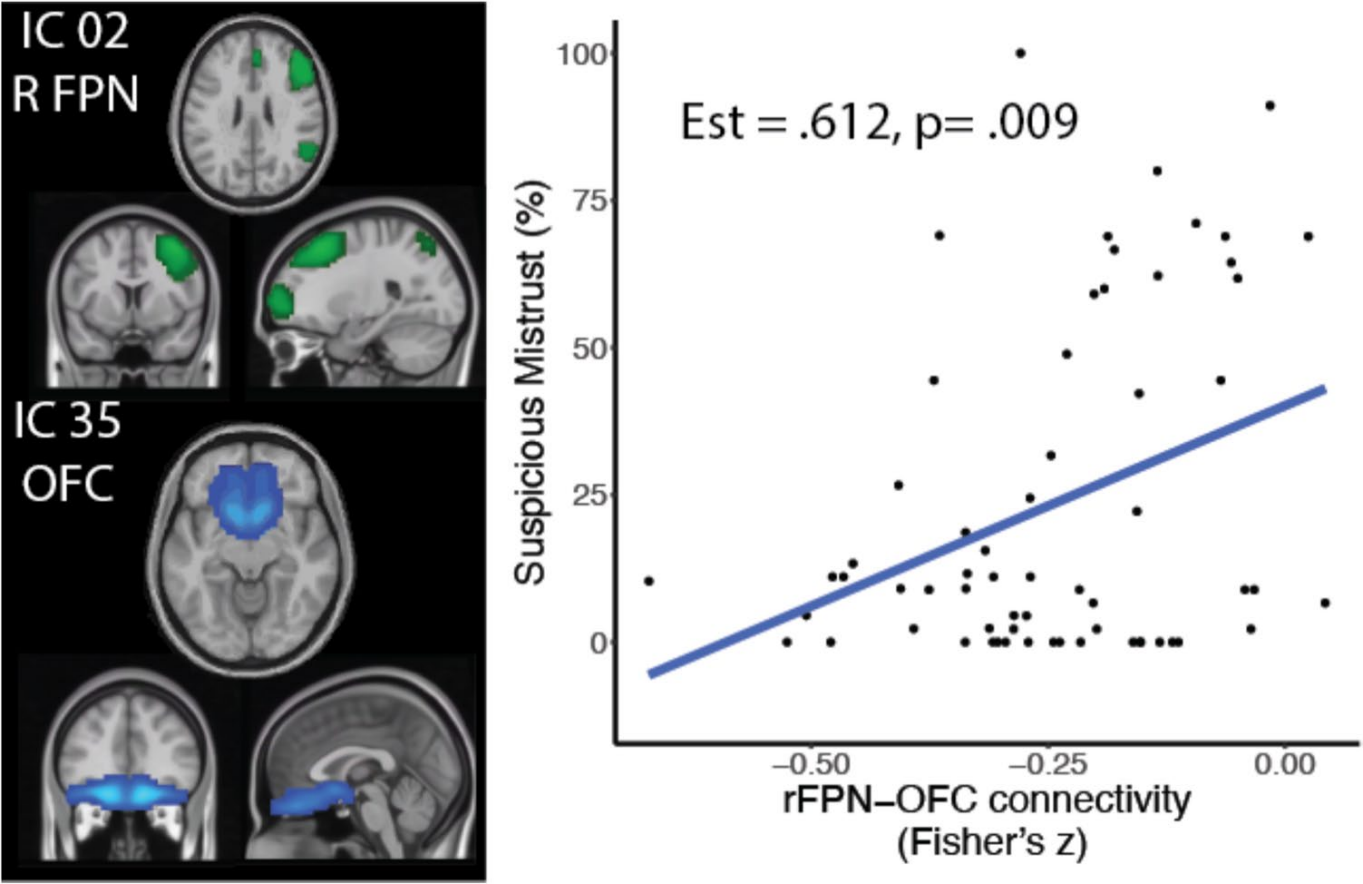
Functional connectivity. Suspicious mistrust was related to the connectivity between the right frontoparietal network and OFC

Lastly, we explored the influence of task performance on functional connectivity analyses. There was a significant positive relationship between right FPN-OFC and suspicious mistrust (estimate = 0.316, SE = 0.117, *t* = 2.71, *p* = 0.009; Fig. 5); spite-guilt beliefs showed a nonsignificant negative relationship for the right FPN-OFC connection (estimate = − 0.240, SE = 0.124, *t* = 1.93, *p* = 0.058). We previously reported that this connection was positively correlated with paranoid thoughts in individuals with psychosis during rest (49).

## Discussion

This study contributes to the growing literature supporting spite sensitivity as a construct underlying suspiciousness. Forty-six community participants (probands) were selected because their MPQ-Alienation scores closely matched those of a group of individuals with psychosis. In addition, 23 of these probands were compared to their monozygotic co-twins. MTG behavior in the community sample replicated previous results for both the first and second mover games. Spite-guilt beliefs and twin differences in spite-guilt beliefs predicted persecutory ideation. In the Rational Mistrust condition, we replicated the relationship with bilateral caudate; when comparing high-risk vs. no-risk trials, left caudate was also associated with risk aversion. Furthermore, right lateral OFC activation when comparing the two conditions was correlated with suspicious mistrust. Bilateral OFC and dmPFC activation comparing high and no risk trials in the Suspiciousness condition predicted suspicious mistrust as well. Differences in trust and no trust decisions in the bilateral OFC differed based on spite sensitivity; representations of subjective value of the chosen outcome in these areas predicted persecutory ideation. When examining twin differences, the left lateral OFC was also associated with suspicious mistrust. These results suggest a potentially causal relationship between lateral OFC activation and spite sensitivity and, in turn, between spite sensitivity and persecutory ideation.

Similar to previously published work (Johnson et al., 2009; Kazinka et al., 2022; 2024; Wisner et al., 2021), we found a relationship between the Suspiciousness condition and persecutory ideation. A recent meta-analysis showed that individuals with psychosis and clinically high-risk participants, but not relatives of individuals with psychosis, had lower baseline trust than healthy controls in the standard trust game (Prasannakumar et al., 2022). This meta-analysis also examined the relationship between positive and negative symptoms and trust but found mixed evidence that positive symptoms (such as delusions) were specifically related to distrust. A key component of the MTG is distinguishing situations in which the second mover is or is not incentivized to betray the first mover, which may explain why we find more consistent results associating persecutory ideation and suspicious mistrust. For example, reported persecutory ideation in a large online sample was associated with increased beliefs that a second mover in the standard trust game was operating with a harmful intent rather than a selfish intent (Greenburgh et al., 2019), consistent with the distinctions of the Suspiciousness and Rational Mistrust conditions. This further suggests that spite sensitivity is a valuable construct, measurable across several samples, that can contribute to our understanding of specific influences on persecutory ideation.

Furthermore, computational modeling linked spite-guilt beliefs and MPQ-Alienation more strongly using the co-twin control approach. Of note, several other persecutory ideation models based on behavioral paradigms exist. Using a modified Social Value Orientation paradigm, Barnby et al. (2022a) developed a model integrating Bayesian modeling and the Fehr–Schmidt inequity aversion model; like our model, guilt was allowed to be negative and was used to predict beliefs of a partner (Barnby et al., 2022a). Barnby & colleagues (2022b) found that individuals with greater paranoia were more likely to want to earn more than their partners, believe that their partners had harmful intent, and have more rigidity in their belief-updating. Similarly, individuals playing a modified Dictator Game showed that greater paranoia was associated with beliefs that their partners had greater harmful intent and more rigidity in these beliefs (Barnby et al., 2022b). In an advice-taking task in a probabilistic lottery game, individuals with persecutory ideation were less likely to trust advice and believed that it was intentionally misleading; hierarchical Bayesian modeling showed that these individuals had more rigid belief-updating (Diaconescu et al., 2020; Wellstein et al., 2020). In contrast, research using a probabilistic reversal learning paradigm, without any social cues, also showed that paranoia is associated with an increased prior belief of volatility in the game in individuals with psychosis, in online samples, and in rats with chronic methamphetamine exposure (Reed et al., 2020; Sheffield et al., 2022). Given that there was no feedback provided in our game, we measure prior beliefs about a partner’s intentions. The use of the co-twin control model provides stronger support that there is a potentially causal link between persecutory ideation and prior beliefs that a partner might not have good intentions. Overall, these models converge on the idea that prior beliefs are more negative and that they remain more rigid despite new information. However, it is unclear if these findings are driven by specifically social priors or more general beliefs about volatility in the environment. Previous work in the MTG showed that spite-guilt beliefs were not associated with performance against a nonhuman random partner (Kazinka et al., 2022), suggesting a privileged role for social information processing.

To further elucidate the mechanisms of spite sensitivity, we examined the interaction of risk and condition on the brain. Our community sample had a more tempered response during decision-making than did individuals with psychosis (Kazinka et al., 2024). Most notably, the whole-brain analysis did not show significant differences between high and no risk trials in the Suspiciousness condition; however, when we examined a priori ROIs more specifically, those with higher spite sensitivity had greater activation in the no risk trials compared to the high risk trials. This result suggests that these regions are still relevant for processing perceived risks seen in the community sample, although to a lesser degree than the individuals with psychosis. Instead, we found that the caudate nucleus activation was greater in the no risk trials compared to high risk trials, similar to the individuals with psychosis previously reported. The caudate nucleus is associated with reward processing (Bartra et al., 2013), and this result may reflect expected processing—in no risk trials, the participants are more likely to trust their partner and would receive a larger amount of money than the safe amount. Previous studies of the standard trust game found reduced activation in the right caudate in individuals with psychosis and nonaffected siblings compared to healthy controls when making investments (Gromann et al., 2013; 2014). Our results suggest intact reward processing in the caudate, although we did not directly compare these findings to another control group. Unlike Gromann & colleagues (2013), the caudate was not associated with persecutory ideation. The MTG sees more specific relationships with persecutory ideation in the Suspiciousness condition instead, while the left caudate activation in the Rational Mistrust condition was associated with risk aversion and concerns with losing money. In comparison to the previously reported individuals with psychosis, our community sample may be able to compensate more readily, showing a more tempered neural response.

We additionally found supporting evidence for neural correlates of spite sensitivity. Suspicious mistrust was associated with lateral OFC when comparing conditions, as well as comparing risk in the Suspiciousness condition. Twin differences in left lateral OFC activation also predicted suspicious mistrust, providing stronger evidence that these factors are related. Further examination of the lateral OFC found that activation was related to the chosen outcome (either trust or not trust), which varied related to suspicious mistrust. However, when we examined the subjective value, the lateral OFC result only appeared in the right lateral OFC, not left. Past research has shown that the lateral OFC encodes hypothetical outcomes (Abe & Lee, 2011) and making inferences about outcomes (Wang et al., 2020). Furthermore, the OFC influences outcome-guided behavior (representations of specific expected outcomes) rather than value-guided behavior (Schoenbaum et al., 2011). Others have demonstrated that the lateral OFC is involved in integrating priors with current information to update beliefs about potential outcomes (Nogueira et al., 2017). This brain region is also associated with assessment of punitive outcomes and nonreward (Rolls, 2016). Together, these results suggest that in the MTG, the lateral OFC is estimating the potential outcomes of the choices, including weighing the potential for punishment or nonreward from the partner. Individuals who believe that their partner will be spiteful may incorporate these contexts into their valuation of each option. However, it is important to note that the lateral OFC has been implicated in emotion processing as well, including perception of and inhibitory control of emotion (Hooker & Knight, 2006), and has been associated with heightened threat perceptions in people with psychosis (Perez et al., 2015). Beliefs about threat or emotional processing may also influence the valuation of these potential outcomes. Some evidence suggests that lateralization in function exists in the lateral OFC, in which value-related processes occur in the left lateral OFC, while emotional processes are more associated with the right lateral OFC (Lopez-Persem et al., 2020). This research also suggests conflicting evidence that lateralization may play distinct roles in social processing as well. We found differences in the pattern of activation in the left and right lateral OFC, and that the left but not right side demonstrated that twin differences predicted behavior, supporting a potential role in different functions.

The lateral OFC was identified as important for suspiciousness decision-making previously in a group of individuals with psychosis and therefore may suggest that altered activation may still occur depending on individual beliefs about spite (Kazinka et al., 2024). These individuals with psychosis also showed that persecutory ideation was predicted by dysconnectivity between the left frontoparietal network and the salience network, including the lateral OFC. It is possible that while emotional processing influences perceptions and valuation of possible outcomes, regulation from the frontoparietal network may further balance these perceptions. In people with psychosis, this reduction in connectivity may be a failure point in regulating the estimation of potential outcomes in the MTG. These results suggest that the lateral OFC is implicated in spite sensitivity.

There are several limitations to this study. Most notably, we had a relatively small sample of monozygotic twin pairs, limiting the power we had to identify potentially causal relationships. Our sample may also be limited due to the influence of trait anxiety on participation in fMRI studies (Charpentier et al., 2021). It is quite possible that higher persecutory ideation also translates into lower trust in science and scientists, and thus a lower probability of participating in fMRI studies. While we targeted elevated persecutory ideation, which may also relate to trait anxiety, our sample may have missed participants at the higher range of persecutory ideation. Nonetheless, we believe that these preliminary results warrant further research in the causal influences of persecutory ideation. In addition, by controlling for shared genetic and experiential influences, the co-twin model offers stronger support for causal associations between variables, but without longitudinal data, the direction of these relationships is unclear. It is also possible that associations may be driven by unshared experiences that influence both persecutory ideation and spite sensitivity but were not included here. Lastly, the spite-guilt beliefs values mainly ranged close to zero, suggesting fewer individuals that demonstrated a fear of spite. However, in the original Fehr-Schmidt model, values were limited to a range of 0–1, so these values are still insightful for our study (Fehr & Schmidt, 1999).

Our unique opportunity to investigate persecutory ideation in monozygotic twins demonstrated that spite sensitivity is potentially causal for persecutory ideation, strengthening our understanding of this relationship in individuals with psychosis and in undergraduate students (Kazinka et al., 2022; 2024). Furthermore, we found that twin differences in left lateral OFC activation also predicted spite sensitivity; this region of interest was previously identified in individuals with psychosis, providing stronger evidence that it may be important in processing spite sensitivity and persecutory ideation. Given these individual differences in spite sensitivity, future research may benefit from examining longitudinal factors such as previous life stress and social support, which might explain the development of persecutory ideation and psychosis. Spite sensitivity, measured by the Minnesota Trust Game, may therefore be an important component in understanding persecutory ideation in research in psychosis and psychosis risk.

## Supplementary Information

Below is the link to the electronic supplementary material.

Supplementary file1 (DOCX 1.68 MB)

## Data Availability

Due to the sensitive nature of the participants’ data, it is not publicly available, but it can be made available upon reasonable request. These hypotheses were derived from previous work (Kazinka et al., 2024; Wisner et al., 2021), but this study was not pre-registered. Unthresholded contrasts from the neuroimaging analyses can be found on NeuroVault: https://neurovault.org/collections/IXZCUDYL/.

## Code availability

The code is available on Open Science Foundation at https://osf.io/3k2z9/.
