# Supplementary material for: Twin differences in the Minnesota Trust Game relate to neural mechanisms of suspiciousness
Source: Cogn Affect Behav Neurosci. 2025 Jul 10;25(6):1711–24. doi: 10.3758/s13415-025-01324-x (PMC12615576; doi:10.3758/s13415-025-01324-x)
Supplement: Supplementary file 1 — Supplementary file1 (DOCX 1.68 MB) [file 13415_2025_1324_MOESM1_ESM.docx]

Supplementary Materials for Twin differences in Minnesota Trust Game relates to neural mechanisms of suspiciousness

Rebecca Kazinka, Anita N.D. Kwashie, Danielle Pratt, Iris Vilares, William Iocono, Sylia Wilson, & Angus W. MacDonald III

1. University of Minnesota Medical School, Psychiatry Department, Minneapolis, MN
2. University of Minnesota, Biomedical Engineering Department, Minneapolis, MN
3. University of Minnesota, Psychology Department, Minneapolis, MN
4. Northwestern University, Psychology Department, Minneapolis, MN
5. University of Minnesota, Institute for Child Development, Minneapolis, MN

Corresponding author: [angus@umn.edu](mailto:angus@umn.edu)

# Additional Methods

## Second Mover Game

Players switched roles and played the Second Mover Game as well, where they decided how to split the money for the first mover (an anonymous participant in the study). Participants were told that the first mover had forgone both players getting $10 to allow the participant to decide between two options mirroring the two conditions based on temptation *T* seen in the First Mover Game ($25 or $15). The Selfishness condition, which follows the Rational Mistrust Condition, offers the second mover (participant) $25 and the first mover the adverse payoff (*Ad*) or $20 for both. The Spite condition offers the second mover a choice between $15 and the first mover the adverse payoff or $20 for both, following the Suspiciousness condition. Adverse payoff amounts mirrored those in the First Mover Game (-$15 to $22).

## Computational methods

Previous studies have more details on the development of this model (Kazinka et al., 2022) and its application (Kazinka et al., 2024). Briefly, four equations were used to estimate the four variables that comprise the model, which are all based on the first mover decisions. In equation 1, the subjective value of each possible outcome in the first mover decision is calculated based on the amount of money that each individual could receive, using the Fehr-Schmidt inequity aversion equation (in which value may be modulated based on envy that a partner has more, as well as risk aversion to losing money compared to the safe amount). In options where both participants receive the same amount, the subjective value is equal to the monetary value. Because two of the potential outcomes are actually based on the second mover’s decision, we additionally modeled the subjective value of those outcomes, but in this case estimated the guilt a partner might feel for getting more. Note that in our model, guilt is *not* based on the second mover performance, and it is allowed to be a negative value, which would indicate spite. Next, based on the decisions of the first mover, we estimated the probability that a player believed the partner would choose the temptation ($15 or $25 based on the condition) using a softmax equation, as the first mover did not control the outcome of that choice. Finally, we calculated the subjective value of choosing to trust the second mover (instead of the safe option) by weighting the subjective values of the two choices with the estimated probability that the partner would choose each one. A softmax equation was also applied to estimate the probability of the first mover choosing to trust or not trust. The inverse temperature parameter (in the softmax equation) was the same for both the first mover and second mover.

The purpose of this model was to understand the preferences for each individual, particularly related to beliefs that a partner might be spiteful (spite-guilt beliefs) and beliefs about the risk of losing money (risk aversion). Crucially, this model provides more context to why a participant chose to trust the partner or not based on the different incentives in each decision. Because there is no feedback in the game, the spite-guilt beliefs parameter demonstrates the first mover’s beliefs about a second mover, regardless of how a second mover might actually behave. We included the risk aversion parameter to further separate specific concerns about a partner’s intentions from general concerns about losing money. In the development of this model, we additionally tested a nonhuman partner, which more concretely separated the influence of a partner’s intentions versus the risk of losing money. The results from that study showed that while spite-guilt beliefs & risk aversion are related, they are distinct (Kazinka et al., 2022). The previous model further tested this hypothesis using two general risk aversion parameters: general risk aversion (the difference between the adverse payoff and the safe amount in all conditions) and social risk aversion, which additionally measured the risk in the human partner conditions. As our current study does not include the nonhuman partner, we collapsed the model to only one risk aversion parameter.

## Neuroimaging GLMs

### Continuous adverse payoff

As an alternative to the Risk × Condition GLM reported in the main text, we additionally ran a GLM using the adverse payoff as a continuous variable while still comparing across the two conditions. The model included an explanatory variable of the trials for each condition and an additional parametric regressor of the mean-zeroed adverse payoff offered for each trial for each condition. We examined contrasts of each explanatory variable, SUS15>RMT25 without the regressors, and the contrast of the conditions when accounting for the parametric regressor of the adverse payoffs.

### Decision-related activation

To examine the role of neural activity in MTG decision-making, we separated the decisions to trust or not trust the partner during each condition. Due to the number of participants who chose to trust or not trust for the majority of trials, we only used a subsample of the participants who had at least one choice for each option (trust SUS15, no trust SUS15, trust RMT25, no trust RMT25) for at least two out of three runs. We included 21 probands and 16 cotwins in this analysis; 12 participants (6 probands and 6 cotwins) only used two runs. We did not run the co-twin control model on these analyses given the small sample size. Given the limitations of our sample size for these analyses, these results should be interpreted with caution.

# Results

## Additional Behavioral Results

### Demographics

To assess sample similarities, we compared the demographics of the probands and co-twins and did not find any significant differences on any characteristics (Table S1). To further assess bias in recruitment, we compared the demographics of probands who had co-twin participation versus not and found only that there were more left-handed paired vs. unpaired probands (*t*(44) = 2.11, *p* = .043). No other differences were found.

### First Mover

An aggregate of individual performances can be found in Figure S1A, which shows that individuals trusted more in the Suspiciousness condition, particularly when the risk increased (lower adverse payoff). We used a Heaviside threshold fit to identify individual thresholds between trusting and not trusting during each condition (Figure S1B). There was a 94.6% accuracy of predicting decisions based on the estimated thresholds. Higher thresholds indicated less trust, as it means participants are not willing to take on more risk. We found a bimodal effect in the Suspiciousness condition and the Rational Mistrust condition, in which participants either fully trusted (thresholds around -$15) or only trusted when risk was low ($10, or *S*) (Figure S1C). There was still a relationship between the thresholds in these two conditions (β = .470, SE = .133, *t* = 3.53, *p* = .001). Simulated data based on participants’ estimated parameters demonstrated similar aggregate results (Figure S1D).

Recovery of the parameters ranged from excellent (spite-guilt beliefs: r(67) = .94, p < .001); inverse temperature: r(67) = .91, p < .001) to good (envy: r(67) = .84, p < .001) and fair (risk aversion: r(67) = .56, p < .001; Figure S2). In addition, the spite-guilt beliefs parameter was negatively correlated with risk aversion (β = -.340, SE = .142, t = 2.39, p = .021) and inverse temperature (β = .329, SE = .143, t = 2.30, p = .031). All other correlations were not significant (*p*’s >.236; Figure S3).

### Second Mover

The Second Mover Game tested participants on both their selfishness, measured in the Selfishness condition, and spite, measured by the Spite condition. Like previous studies, selfishness decreased as the adverse payoff decreased in the Selfishness condition, but almost no one chose to be spiteful (temptation × adverse payoff: β = .134, *Z* = 2.28, *p* = .022; Figure S4A). The main effects of temptation and adverse payoff were also significant (*p*’s < .001). Like the First Mover Game, a Heaviside threshold was fit to each individual (predicting 94.4% of choices), which revealed a bimodal effect in the Selfishness condition with the majority of individuals choosing to be completely selfish; in contrast, the majority chose to be completely fair in the Spite condition. There was no correlation between the two conditions (β = .124, SE = .151, *t* = .819, *p* = .42; Figure S4B).

To further examine selfishness in the Second Mover Game, we compared participants based on low and high empathic concern from the Interpersonal Reactivity Index (IRI; Davis, 1980). We found a significant interaction of temptation × adverse payoff × empathic concern, such that those who were lower on empathic concern were more selfish in the Selfishness condition, replicating previous results (β = .153, *Z* = 2.33, *p* = .020; Figure S4C). There was also a significant interaction of temptation and empathic concern, in which differences in empathic concern were only seen in the Selfishness condition (β = -.413, *Z* = 5.69, *p* < .001). A main effect of empathic concern showed that those who scored higher chose the fair option more often (β = -.364, *Z* = 3.18, *p* = .001). The interaction of adverse payoff and empathic concern was not significant (β = .110, *Z* = 1.15, *p* = .250). Twin differences in empathic concern did not predict task performance on the Second Mover Game (*p*’s >.26).

Most importantly, when we compare the two games, we see that while many individuals are concerned about their partners being spiteful in the Suspiciousness condition, almost no one behaves spitefully in the corresponding Second Mover condition (Figure S4D). There is not a significant relationship between the First and Second Mover Games for the Suspiciousness and Spite conditions (β = .006, SE = .151, *t* = .041, *p* = .968). There is also not a correlation between performance in the Selfishness and Rational Mistrust conditions, suggesting that individuals are not simply playing against themselves, but actually imagining a partner (β = -.086, SE = .114, *t* = .756, *p* = .458).

## Additional Neuroimaging results

### Main effect of risk and condition

Figure S5 shows additional *a priori* ROIs from Kazinka et al., 2024. Figure S6 shows the whole brain results of the main effects and interaction of risk and condition of the twins reported in the main text. The interaction between risk and condition showed a small region of positive activation in the caudate but no other frontal regions. When we examined the main effect of risk, we found a significant reduction in activation in bilateral caudate during high risk trials, consistent with previous results; whole brain analysis also showed reduced activation in the right middle frontal gyrus and right supramarginal gyrus, which is partially consistent with the individuals with psychosis. Finally, the main effect of condition did not show significant activation in any of the selected ROIs (Fig. 3A & S5); a whole brain analysis identified greater activation in the Suspiciousness condition in the occipital regions, precuneus, and right frontal pole.  Lastly, when we compared the twins against their cotwins (using a within-subject model) in a whole-brain analysis, we only found a significant difference in the right lateral occipital cortex (superior) when examining the high risk > no risk contrast during rational mistrust condition (Figure S6D). This result is expected, given that there were no significant group differences in MPQ-Alienation.

### Continuous Adverse Payoff

 We ran a GLM examining the adverse payoff as an additional parametric regressor, separated by each condition (Figure S7). The difference between the two condition-related adverse payoff contrasts (SUS15 AD > RMT25 AD; similar to the interaction effect reported in the main text) identified regions of interest in the precuneus, left lateral OFC, and right caudate (Fig. S7A). The precuneus and left lateral OFC were not seen in the main text analysis, but we replicated the right caudate. When looking at the activation related to the adverse payoff in the SUS15 condition, there was a positive relationship in the right parietal cortex and a negative relationship in the left lateral OFC and posterior cortex (Fig. S7B). In contrast, the adverse payoff in the RMT25 condition was positively associated with right caudate, right anterior insula, and right frontoparietal cortex (Fig. S7C). When we extracted the same ROIs used in the main text from Kazinka et al. (2024), there remained a significant correlation between suspicious mistrust and bilateral OFC and dmPFC relevant to the adverse payoff during SUS15 condition (left lateral OFC: β = .400, SE = .111, *t* = 3.59, *p* < .001; right lateral OFC: β = .324, SE = .108, *t* = 2.99, *p* = .005; dmPFC: β = .300, SE = .110, *t* = 2.74, *p* = .008; Fig. S7D). Twin differences in lateral OFC activation also predicted suspicious mistrust, mirroring results in the main text (β = .258, SE = .087, *t* = 2.98, *p* = .005; Fig. S7E). While these are positive relationships compared to the negative relationships in the main text, this result is consistent because, in the main text, higher risk (i.e., lower adverse payoff) is associated with higher values.

### Decision-related activation

To better understand the neural mechanisms behind the decision to trust, we compared activation during trust and no trust trials for each condition (Figure S8). When comparing trust > no trust trials across conditions, we found positive activation in bilateral OFC, right inferior temporal gyrus, occipital lobe, and superior parietal gyrus. We found that trust > no trust in the RMT25 condition showed right lateral OFC. In contrast, there were no significant regions of interest when comparing trust > no trust in the SUS15 condition. When we lowered the threshold for clustering (z = 2.3), we found greater activation during trust > no trust during the  SUS15 condition in bilateral OFC, bilateral inferior temporal lobe, occipital lobe, and superior parietal gyrus.

We further examined the activation of the three ROIs described in the main text (bilateral OFC and dmPFC). Activation in the left lateral OFC comparing trust and no trust decisions showed a pattern in which those with lower suspiciousness thresholds (suggesting low spite sensitivity) had more negative activation in the left lateral OFC in response to the trust decision in the SUS15 condition, but in contrast they had more negative activation in no trust decisions for the RMT25 condition. For individuals who had higher suspiciousness thresholds (suggesting high spite sensitivity), there was more negative activation in the left lateral OFC in the no trust trials for both conditions (SUS15 threshold × choice × condition 3-way interaction: β = .663, SE = .210, *t* = 3.16, *p* = .004). There was a significant SUS15 threshold × condition effect, in which there was more negative activation during the SUS15 condition compared to RMT25 condition for those with higher thresholds (β = -.414, SE = .148, *t* = 2.79, *p* = .009). There was also a significant choice × condition interaction, in which there was more negative activation in the left lateral OFC during no trust choices compared to trust choices in the RMT25 condition, but no difference between the two choices for the SUS15 condition (β = -.504, SE = .209, *t* = 2.40, *p* = .023). Lastly, there was a main effect of trust, with more negative activation in the no trust trials (β = .516, SE = .148, *t* = 3.49, *p* = .002). Activation in the left lateral OFC during no trust SUS15 trials was correlated with spite-guilt beliefs (β = .337, SE = .158, *t* = 2.13, *p* = .043) and suspicious mistrust (β = .336, SE = .158, *t* = 2.13, *p* = .043), but not persecutory ideation (β = -.185, SE = .164, *t* = 1.13, *p* = .275). We hypothesized that the left lateral OFC is representing anticipation of the chosen outcome.

For the right lateral OFC, we found that those with low SUS15 thresholds had a more positive activation during trust choices in RMT25 condition, but more positive activation during no trust choices in the SUS15 condition. In contrast, those with high SUS15 thresholds had negative activation associated with no trust choices during the SUS15 condition, with a significant 3-way interaction (SUS15 threshold × choice × condition 3-way interaction: β = .673, SE = .208, *t* =3.24, *p* = .003). There was a significant interaction of SUS15 thresholds and condition, in which those with low thresholds (more trusting) had more positive activation in both conditions, while those with higher thresholds (less trusting) had negative activation, particularly in the SUS15 condition compared to the RMT25 condition (β = -.35, SE = .147, *t* = 2.39, *p* = .024). There was also a main effect of trust, in which more positive activation was seen in trusting choices (β = .308, SE = .146, *t* = 2.11, *p* = .045). Activation in the right lateral OFC during no trust SUS15 trials was correlated with spite-guilt beliefs (β = .562, SE = .157, *t* = 3.6, *p* = .001) and suspicious mistrust (β = -.608, SE = .147, *t* = 4.15, *p* < .001), but not persecutory ideation (β = -.100, SE = .186, *t* = .526, *p* = .603).

For the dmPFC, there were minimal differences related to SUS15 thresholds. There was a choice × condition effect, in which there was more activation in trust over no trust trials in the SUS15 condition only (β = .599, SE = .264, *t* = 2.27, *p* = .031). There was also a main effect of condition (β = -.445, SE = .187, *t* = 2.38, *p* = .024). There were no significant correlations with spite-guilt beliefs, suspicious mistrust, nor persecutory ideation *p’s* > .088.

### Functional connectivity

ICNs are shown in Figure S9. Unlike individuals with psychosis previously reported, the connectivity between right frontoparietal network (FPN) and OFC/insula/dmPFC was *not* predicted by MPQ-Alienation scores (β = -.105, SE = .128, *t* = .823, *p* = .415) nor was left FPN-OFC/insula/dmPFC connectivity (β = .079, SE = .117, *t* = .677, *p* = .502). Left FPN-OFC connectivity also was not predicted by MPQ-Alienation scores (β = -.037, SE = .128, *t* = .286, *p* = .776). Instead, MPQ-Alienation predicted the connectivity between caudate and dmPFC (β = -.249, SE = .119, *t* = 2.10, *p* = .041). While unpredicted, this result may suggest that the caudate nucleus plays a more significant role in the task in our community sample. Unlike the individuals with psychosis, we did not see a relationship between OFC/insula/dmPFC-left FPN and the likelihood of mistrust (β = -.068, SE = .068, *t* = .565, *p* = .575). Overall, there were fewer relationships between functional connectivity and persecutory ideation in our community sample.

### Neuroimaging twin differences

Suspicious mistrust was not predicted by twin differences in right lateral OFC or dmPFC during the suspiciousness condition when comparing high and no risk (p’s > .135), but mean difference (*i.e.*, between twin pairs) in dmPFC activation was associated with suspicious mistrust (β = -.389, SE =.121, *t* = 3.22, *p* = .004), as well as right lateral OFC (β = -.324, SE = 128, *t* = 2.51, *p* = .019). In the Suspiciousness > Rational Mistrust contrast, we did not find that twin differences in right lateral OFC spite-guilt beliefs (β = -.153, SE = .119, *t* = 1.28, *p* = .214) nor suspicious mistrust (β = .119, SE = .098, *t* = 1.21, *p* = .236). In terms of functional connectivity, right FPN-OFC was predicted by between-twin pair differences in suspicious mistrust (β = .345, SE = .121, *t* = 2.86, *p* = .009), but not within-twin pair differences (β = .104, SE = .121, *t* = .861, *p* = .398). A similar pattern emerged when examining right FPN-OFC connectivity, which was predicted by between-twin pair differences in spite-guilt beliefs (β = -.322, SE = .122, *t* = 2.64, *p* = .015), but not within-twin pair differences (β = .049, SE = .012, *t* = .402, *p* = .691). These between-pair differences are more consistent with potential shared liability (*i.e.,* genetic and shared environmental factors).

# References

Davis, M. H. (1980). *Interpersonal Reactivity Index (IRI)*. APA PsycTests. [https://doi.org/10.1037/t01093-000](https://psycnet.apa.org/doi/10.1037/t01093-000)

Kazinka R, Vilares I, MacDonald III AW. A Computational Model of Non-optimal Suspiciousness in the Minnesota Trust Game. Comput Psychiatry. 2022;6(1):60–78.

Kazinka, R., Kwashie, A. N. D., Pratt, D., Vilares, I., & MacDonald, A. W. (2024). Value representations of spite sensitivity in psychosis on the Minnesota Trust Game. *Biol Psychiatry Cogn Neurosci Neuroimaging.* DOI: 10.1016/j.bpsc.2023.11.010.

Table S1. Demographics by group.

| Category | Probands | Co-Twin | Probands vs. Co-Twins | Proband paired vs. unpaired |
| --- | --- | --- | --- | --- |
| N | 46 | 23 | 69 | 46 |
| Age | 31.1 (5.3) | 30.3 (5.2) | t(44) = .66, p = .513 | t(44) = 1.13, p = .262 |
| % Male | 45.6% | 39.1% | X^2^ = .066, p = .797 | X^2^ = 0, p = 1 |
| Years of Education | 16.6 (2.2) | 16.4 (2.7) | t(44) = .29, p = .773 | t(44) = .94, p = .35 |
| Parental Years of Education (average) | 14.6 (3.2) | 15.3 (3.2) | t(44) = .84, p = .405 | t(44) = 1.78, p = .08 |
| Handedness Laterality | 68.5 (62.5) | 82.9 (39.0) | t(44) = 1.01, p = .318 | **t(44) = 2.11, p = .043** |
| % Racial Minority | 2.2% | 4.3% | X^2^ = 0, p = 1 | X^2^ = 0, p = 1 |
| % Hispanic or Latinx | 2.2% | 0% | X^2^ = 0, p = 1 | X^2^ = 0, p = 1 |
| MPQ-Alienation | 35.4 (7.7) | 32.6 (8.6) | t(44) = 1.38, p = .174 | t(44) = .26, p = .79 |

Note. Parentheses indicate standard deviation. Degrees of freedom were reduced to match the unique number of families in the study (46).

**
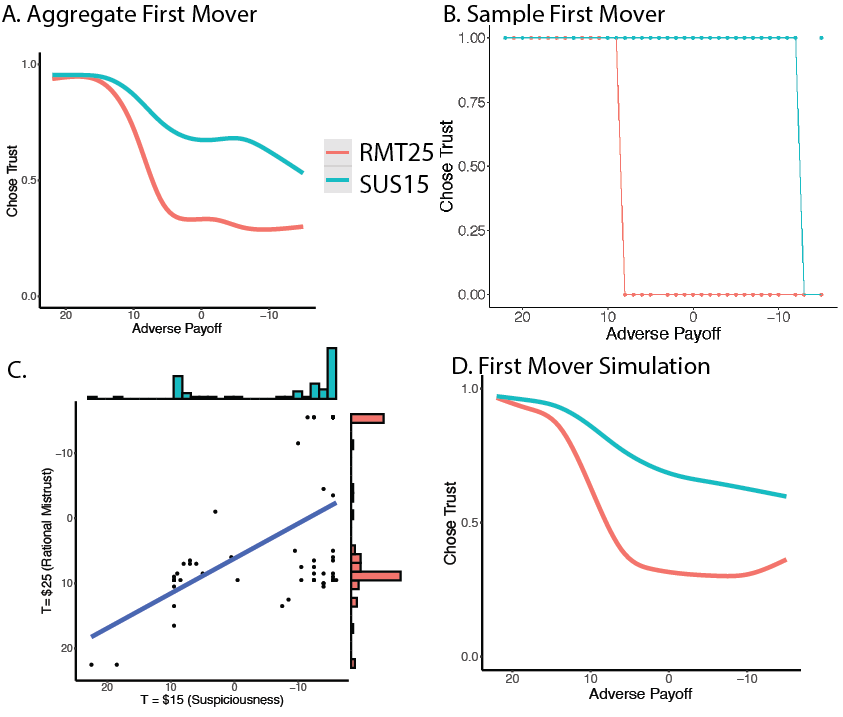
**

**Figure S1. Minnesota Trust Game First Mover Game behavior.** A) Aggregate behavior in the First Mover Game across all participants, divided by condition. Trust decreased when the Adverse payoff decreased (further right of x-axis), but more drastically decreased at $10 (*S*) for the Rational Mistrust condition (RMT25, in red), while it decreased to a lesser degree in the Suspiciousness condition (SUS15, in blue). B) Sample First Mover Game behavior of an individual. Thresholds were calculated based on a Heaviside function for each condition for each participant. C) Comparisons of thresholds showed that most participants had a threshold at $10, although a group also trust even at low adverse payoffs. There was a bimodal effect in the Suspiciousness condition, in which individuals mostly trusted even when payoffs were negative, but others stopped trusting at $10. D) The Spite Sensitivity Model successfully showed similar results when we simulated behavioral data based on individual estimated parameters.

**
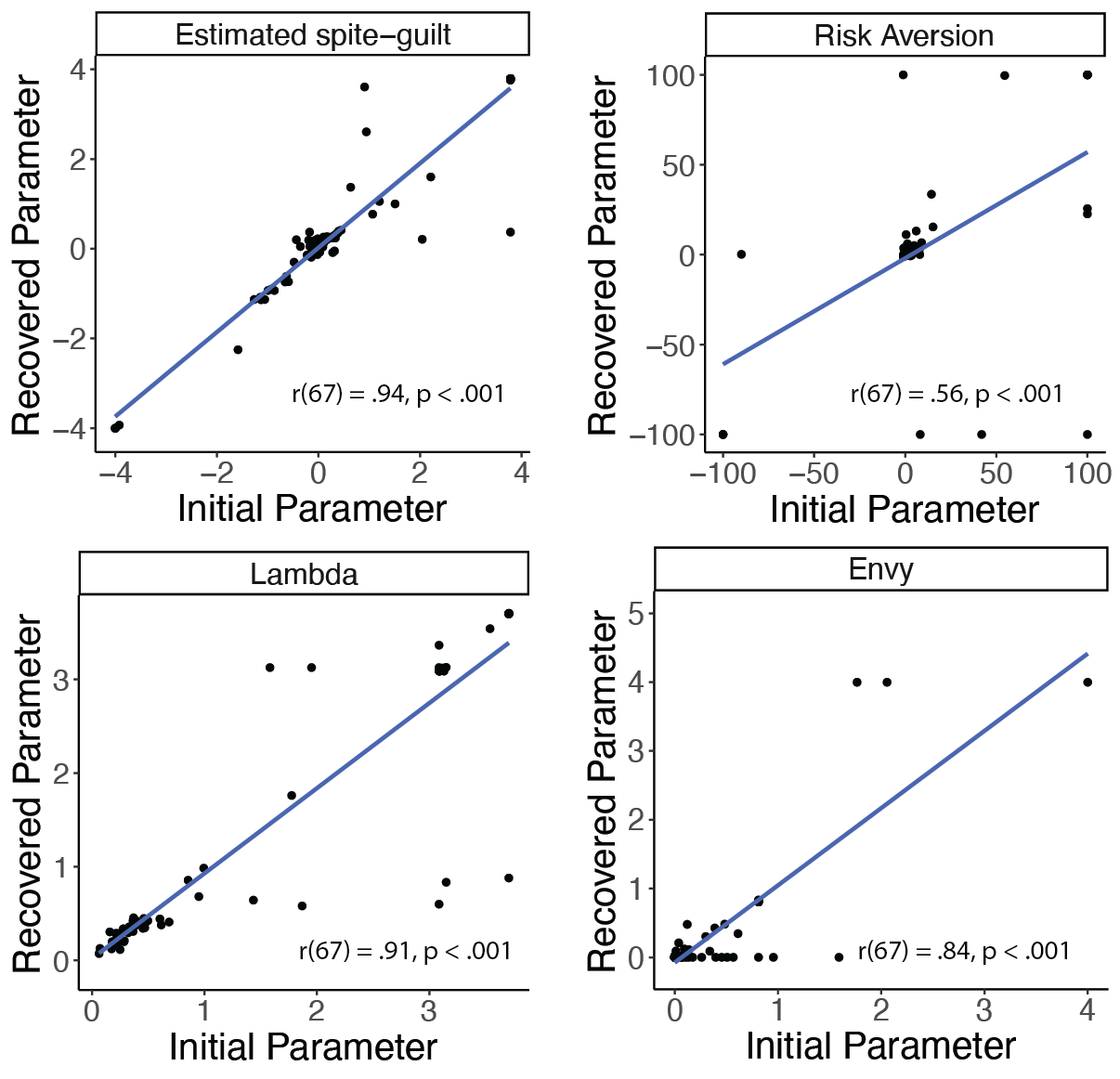
**

**Figure S2. Recovery of computational parameters.** Recovery of parameter estimates ranged from fair (risk aversion) to good (envy), and excellent (estimated spite-guilt and inverse temperature).

**
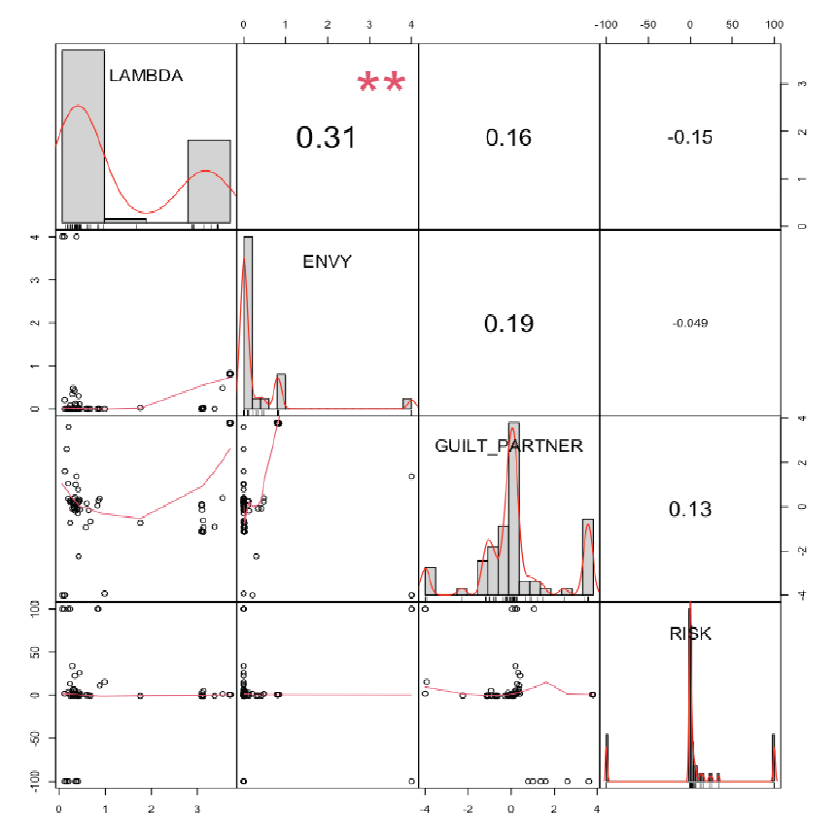
**

**Figure S3. Correlation matrix of parameter estimates.** The diagonal shows the histogram for each parameter. The bottom half shows the scatter plots between each parameter, while the top half reports the Spearman correlation between the parameters. The correlation between lambda (inverse temperature) and envy was significant. Figure created using chart.Correlation in R.

**
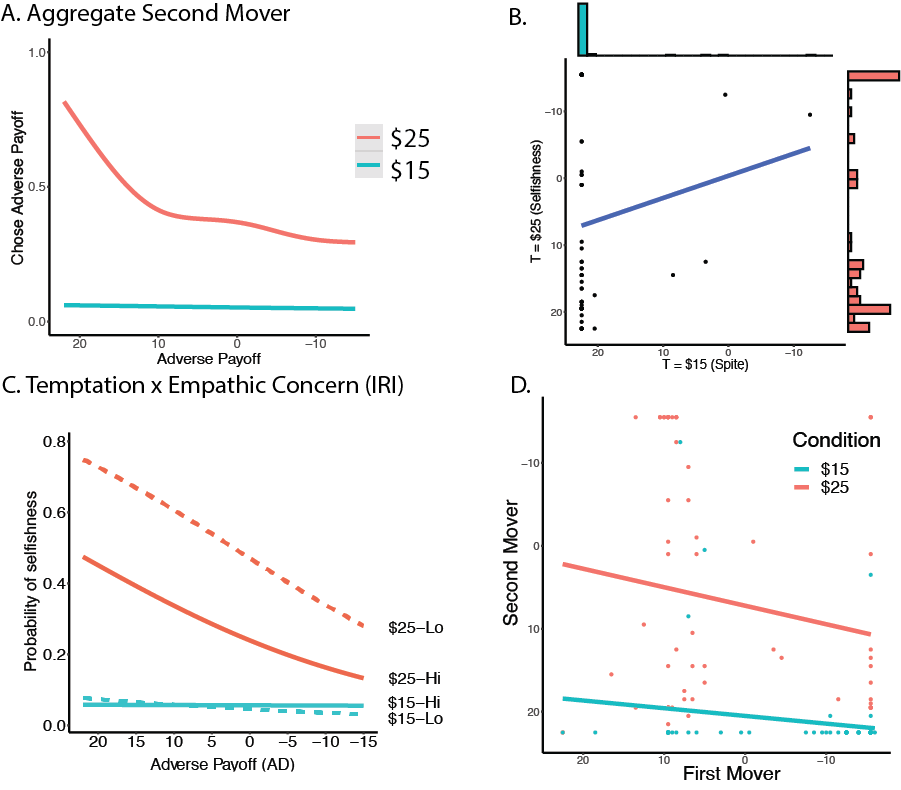
**

**Figure S4. Second Mover Game behavior.** A) Aggregate Second Mover Game behavior showed decreasing selfishness as adverse payoff decreased in the Selfishness condition, and almost no spiteful behavior in the Spite condition. B) There was no correlation between behavior in the two conditions, which showed a bimodal effect of thresholds in the Selfishness condition, but almost all individuals chose the fair option for the Spite condition. C) Those who scored higher on the IRI-empathic concern scale also were less selfish during the Selfishness condition. D) There were not significant relationships between performance on the First Mover Game and Second Mover Game across conditions, suggesting that participants viewed their partners as different from themselves.


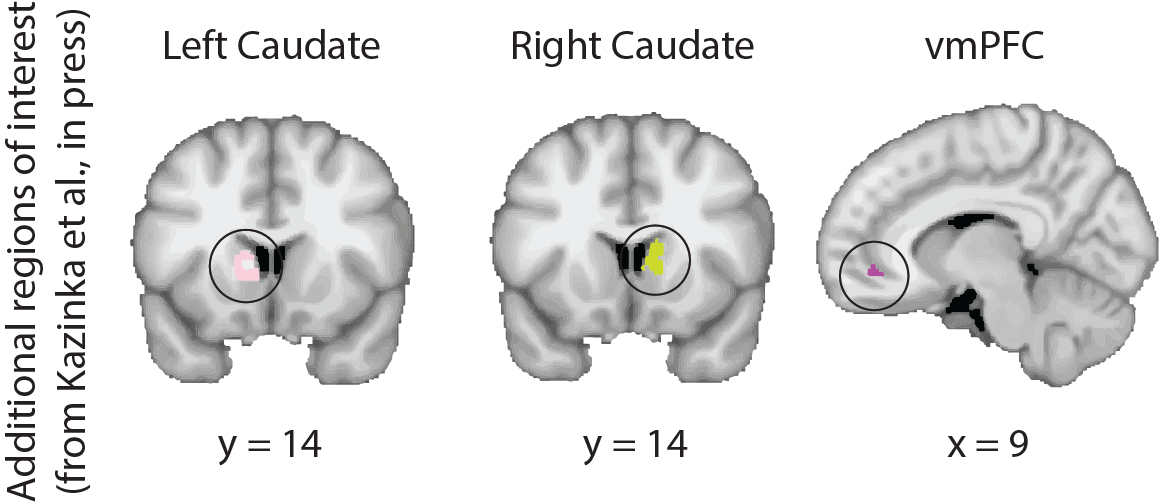


**Figure S5. Additional ROIs from Kazinka et al., 2024.** ROIs were identified based on the interaction of risk and condition in the Minnesota Trust Game from 49 individuals with psychosis. In addition to the bilateral OFC and dmPFC reported in the main text, we also examined the bilateral caudate nucleus and vmPFC.


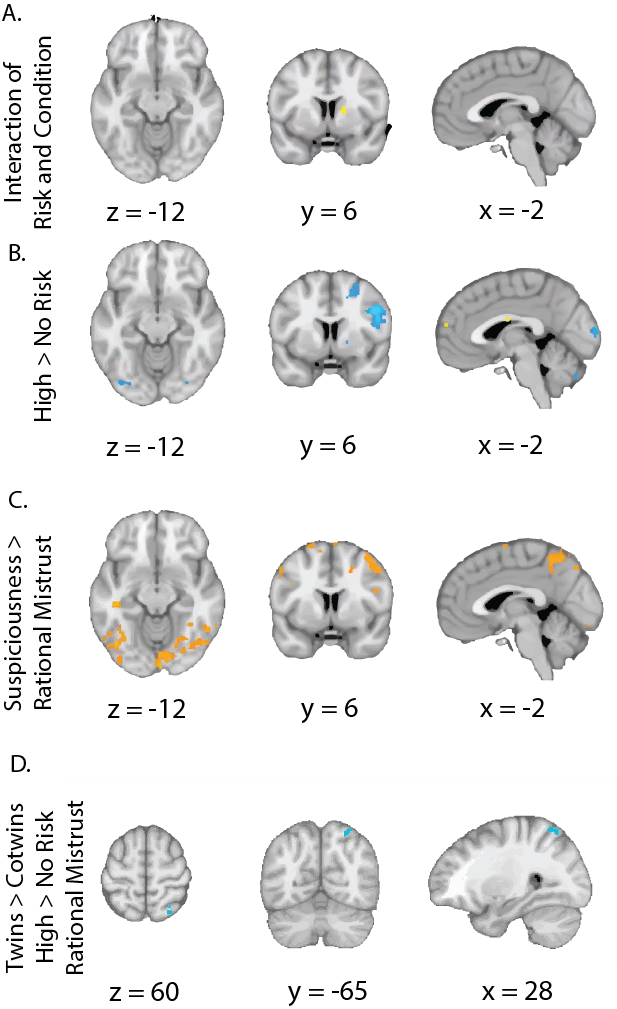


**Figure S6. Generalized Linear Model of Risk** × **Condition.** A) Interaction of risk and condition for the community sample showing activation in the caudate nucleus. B) When comparing high to no risk trials, we found decreased activation during high risk trials in the bilateral caudate. C) There were not any significant changes in activation in the *a priori* ROIs when comparing the two conditions. Instead, we saw greater activation in the Suspiciousness condition in the occipital regions, precuneus, and frontal pole. Orange indicates positive activation and blue indicates negative activation. D) There were no differences between the twins and co-twins except for in the right lateral occipital lobe during high > no risk rational mistrust trials.

**
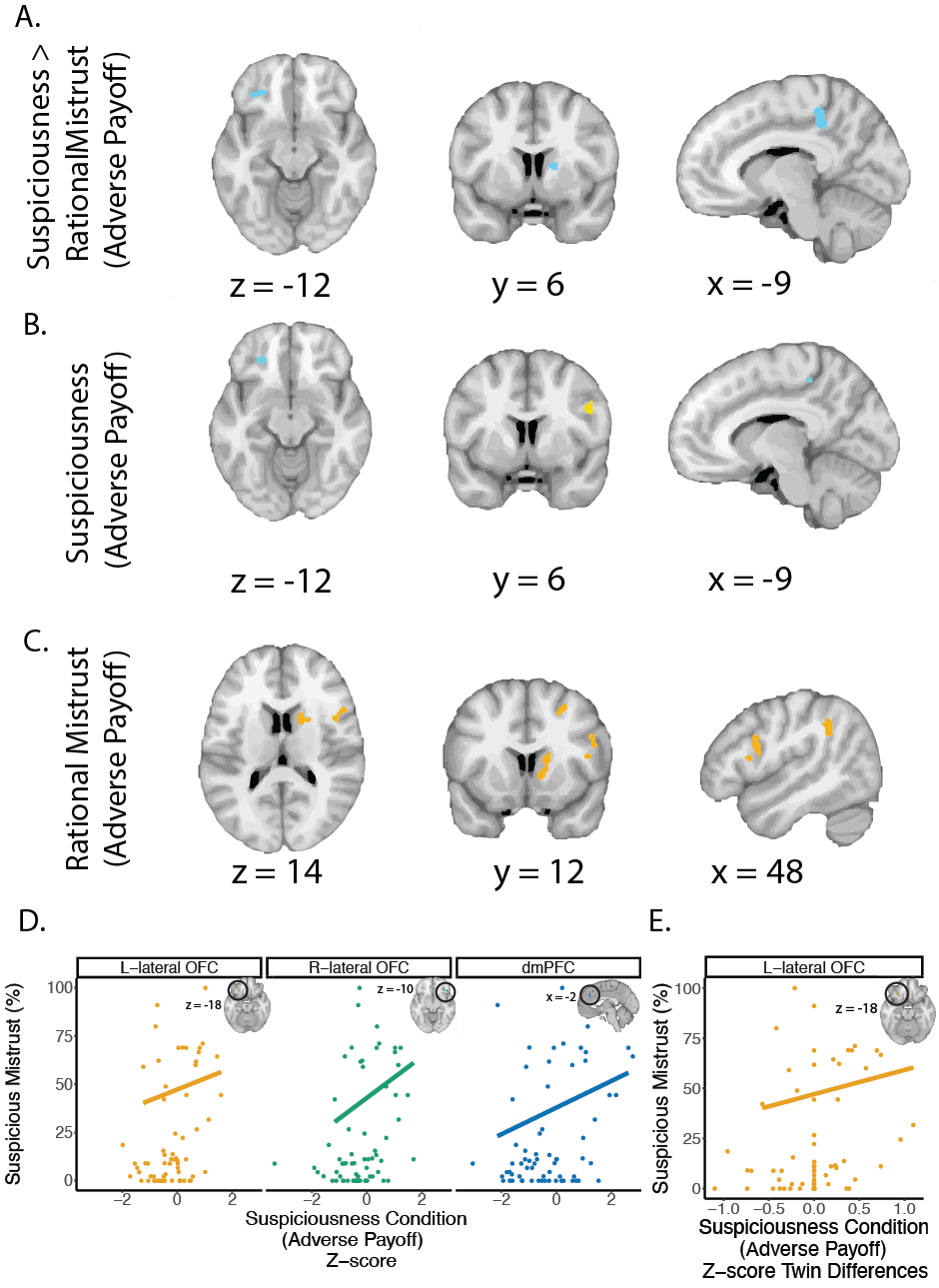
**

**Figure S7. Risk** × **Condition GLM using adverse payoff as a continuous variable.** A. Comparisons of the adverse payoff relationships in the suspiciousness condition over the rational mistrust condition we associated with left lateral OFC, right caudate nucleus, and precuneus. B. When looking at adverse payoff for the suspiciousness condition only, we found it was related to right parietal cortex and left lateral OFC and posterior cortex. C. The adverse payoff activation for the rational mistrust condition showed activation in the right caudate, right anterior insula, and right frontoparietal cortex. D. Activation related to the adverse payoff in the suspiciousness condition for the bilateral OFC and dmPFC was correlated with suspicious mistrust. E. Twin differences in the adverse payoff in the suspiciousness condition for the left lateral OFC also predicted suspicious mistrust.

**
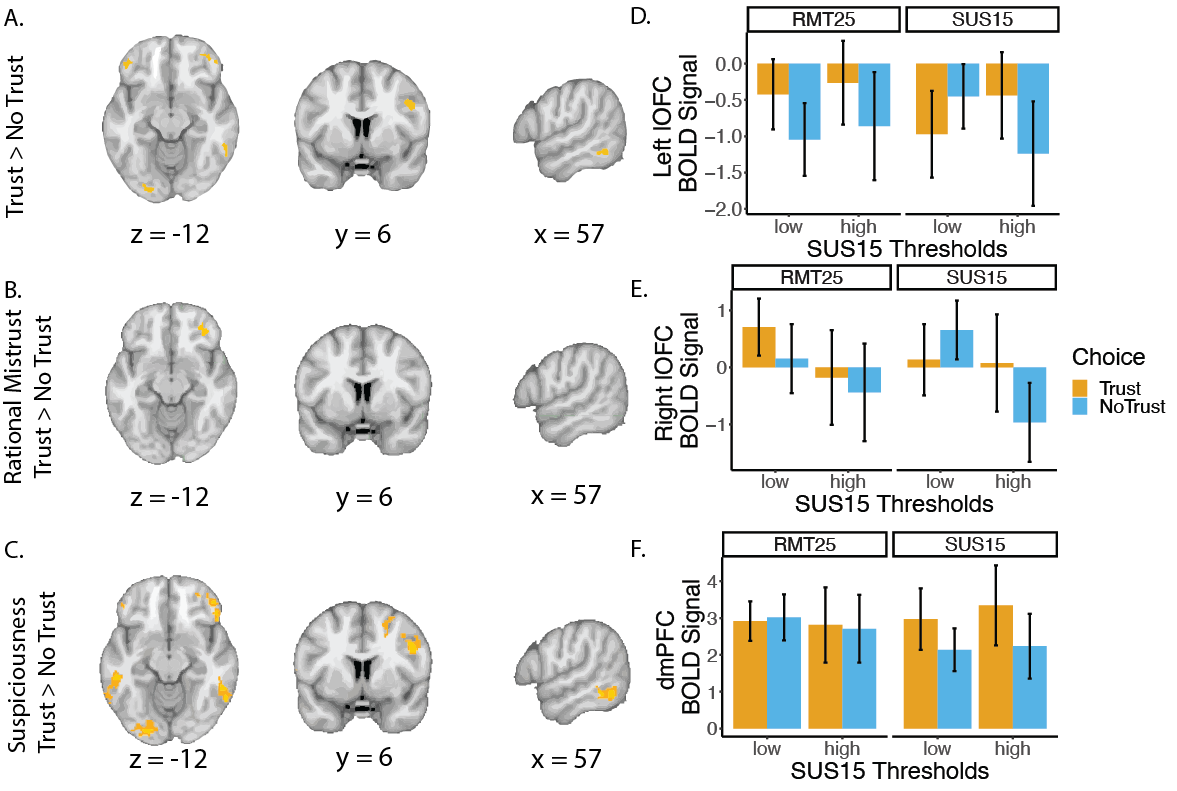
Figure S8. Decision-related activation.** A) Trust > no trust trials were associated with bilateral OFC, right inferior temporal gyrus, occipital lobe, and superior parietal gyrus. B) In the rational mistrust condition, trust > no trust trials were associated with right lateral OFC. C) There were no differences in trust > no trust trials in the suspiciousness condition. However, when we reduced the threshold for clustering (z = 2.3 instead of 3.1), we found activation in bilateral OFC, bilateral inferior temporal lobe, occipital lobe, and superior parietal gyrus. D) Individuals with higher suspiciousness condition thresholds had more negative left lateral OFC activation during no trust trials in the suspiciousness condition, but those with low suspiciousness thresholds had more negative left lateral OFC activation during trust trials in the suspiciousness condition. This difference did not exist in the rational mistrust condition. E) Activation in the right lateral OFC was positive for trust choices in the rational mistrust condition and no trust choices in the suspiciousness condition, but only for individuals with low suspiciousness thresholds (more trusting). Activation was negative for those with high suspiciousness condition thresholds. F) Activation in the dmPFC was positive during decision making, but there were no differences related to suspiciousness condition thresholds.

# **
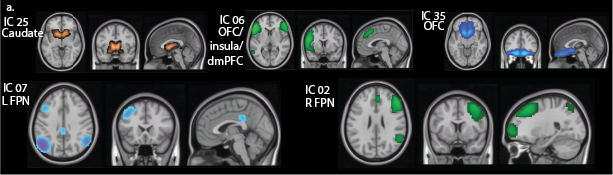
**

**Figure S9. Intrinsic Connectivity Networks.** Selected *a priori* independent components selected from a community sample (Reuter et al., 2018). This includes the caudate nucleus, OFC/insula/dmPFC, orbitofrontal cortex (OFC), and left and right frontoparietal networks (FPN). Adapted from Kazinka et al. (2024) and used with permission.
